# Factors Associated with Health Service Use for Self-Reported Balance Problems in Community-Dwelling Adults: A Secondary Analysis of Nationally Representative NHANES 2001–2004 Data

**DOI:** 10.3390/healthcare13202654

**Published:** 2025-10-21

**Authors:** Shweta Kapur, Kwame S. Sakyi, Joshua L. Haworth, Prateek Lohia, Daniel J. Goble

**Affiliations:** 1 Department of Human Movement Science, Oakland University, Rochester, MI 48309, USAdgoble@oakland.edu (D.J.G.); 2 Department of Public and Environmental Wellness, Oakland University, Rochester, MI 48309, USA; 3 Department of Internal Medicine, Wayne State University, Detroit, MI 48202, USA

**Keywords:** balance, falls, healthcare utilization, healthcare, NHANES

## Abstract

**Background:** Balance problems are one of the major risk factors for falls. Despite the availability of effective fall prevention interventions, falls and related injuries are rising. This study explored the factors associated with healthcare utilization for balance problems in community-dwelling adults in the United States. **Methods:** Study involved secondary analysis of nationally representative National Health and Nutrition Examination Survey 2001–2004 data (latest data with variables of interest at the time of study). All adults (≥40 years) who reported balance problems in the past 12 months were included. Dependent variable was whether the individual ever saw a healthcare professional for balance problems. All analyses were adjusted for probability sampling weights. Adjusted odds ratio (AOR) and 95% confidence interval (CI) were calculated using multivariable logistic regression. **Results:** Study included 1834 adults with self-reported balance problems (mean age 60.1 years (0.5 SE), 62.3% females). Of these, only 32.13% ever saw a healthcare professional for their balance problems. Having encounter(s) with a healthcare provider for any reason in the past year (AOR 2.45; 95% CI,1.19–5.06; *p* = 0.017), lack of health insurance (AOR 0.52; 95% CI,0.32–0.84; *p* = 0.009), falls in the past year (AOR, 1.29; 95% CI,1.03–1.61; *p* = 0.028) and age (AOR, 0.98; 95% CI,0.97–0.996; *p* = 0.011) had significant association with healthcare utilization for balance problems. The predicted probability of healthcare use for balance problems decreased from 0.39 for 40-year-olds to 0.26 for 80-year-olds. **Conclusions:** This study reports the association between factors such as age, health insurance, encounter with a healthcare provider, and falls in the past year with healthcare utilization for balance problems among community-dwelling adults with self-reported balance problems and identifies populations at increased risk of underutilization. Despite the use of older data, it provides useful information for guiding future research in this novel domain of healthcare research.

## 1. Introduction

Balance problems are highly prevalent in older adults [1]. These balance problems can result in falls, which are the leading cause of mortality and morbidity in community-dwelling adults [2]. Balance problems have also been associated with an increased risk of mortality from all causes, in individuals aged 40 years or more [3]. The existing literature supports the efficacy of exercise and multifactorial interventions for fall prevention [4,5,6,7]. Despite efforts at different levels to minimize falls, the number of falls, related injuries, and their burden on the healthcare system is rising [1,8]. Given the trend of increasing falls and related injuries despite the availability of effective fall prevention interventions, it is crucial to determine how many people with balance problems are actually utilizing these interventions. Relatedly, it is important to identify the factors that prevent individuals from accessing them.

There is a scarcity of literature exploring predictors of healthcare utilization for balance problems, especially for individuals in the fourth and fifth decades of life. The Andersen Healthcare Utilization Model [9,10], conceived in 1960s, is one of the most widely used frameworks for examining factors influencing the use of health services. It categorizes these factors into three groups: predisposing factors, enabling and disabling factors, and factors affecting the need for care [10,11]. A recent literature review [12] used this model to examine potential factors associated with the underutilization of healthcare services for managing balance problems in community-dwelling adults. The review reported factors such as age, sex, race, body mass index, and preexisting comorbidities that predisposed individuals to the underutilization of healthcare services for balance problems. Access to a primary care provider, health insurance coverage, and socioeconomic status could enable or disable healthcare utilization. It was also posited that the occurrence of falls, individual’s perception of the balance problem, and its impact on activities of daily living can affect the need for healthcare utilization [12]. Further review of the literature also suggests that individual’s mental health status, marital status, and physical activity levels may also impact the use of healthcare services. An increased prevalence of balance problems and falls has been reported in individuals with mental health issues [13,14]. It has also been indicated that healthcare utilization can be higher in individuals with balance problems who have associated anxiety and depression [15]. Reduced use of inpatient and skilled nursing facilities and increased use of outpatient services has been reported in married individuals compared to those who are unmarried [16]. Increased physical activity levels have been associated with decreased healthcare utilization [17]. However, there is a need for confirming these potential associations indicated by the literature using real world data [12].

Despite inconsistent differences between males and females on different performance-based balance tests reported in the literature [18,19,20], there is a higher incidence of falls in females [8]. Age-related loss of bone mineral density is greater in females than males [18]. Females have also been reported to be more willing to engage in interventions to improve balance after experiencing falls [21]. This aligns with reports of higher healthcare utilization for fall-related injuries among females, especially older females compared to older males [8]. These sex differences in healthcare utilization are further evident in those with preexisting comorbidities [22,23,24]. Hence, it seems worthwhile to explore if sex is an effect modifier for the association between healthcare utilization for balance problems and other factors.

Previous literature has explored healthcare utilization for falls and fall-related injuries [8,25], but there is no known study exploring the factors associated with healthcare utilization for balance problems at the population level. It is both important and challenging to identify individuals for balance-related healthcare services [7]. Identifying factors that prevent or reduce the utilization of healthcare services for balance problems can guide future efforts to improve access to effective interventions for vulnerable populations. Therefore, further research using real-world population-level data, such as the National Health and Nutrition Examination Survey (NHANES) [26,27,28], is needed.

The current study explores the factors associated with healthcare utilization for balance problems in community-dwelling adults in the United States of America (US). This was an exploratory study for hypothesis generation, which aims to lay the groundwork for future research that can validate and expand upon these results. This study further provides insight into age-sex, sex-falls, and sex-comorbidity interactions for healthcare utilization for balance problems.

## 2. Significance

Knowledge about the factors influencing healthcare utilization for balance problems in community-dwelling adults in the US can help both the researchers and regional/national organizations convene, integrate, and influence healthcare policies promoting health equity by improving access to healthcare for vulnerable populations. Timely management of balance problems, before they result in a serious event, can help improve patient outcomes and reduce the burden on the healthcare system.

## 3. Methods

### 3.1. Research Questions

The research questions for the study were what proportion of the US population (aged 40 years or more) with self-reported balance problems sought healthcare services for balance problems; and what factors influenced the healthcare utilization for balance problems among individuals with self-reported balance problems.

### 3.2. Study Design

This study involved a secondary analysis [26] of the data from the National Health and Nutrition Examination Survey (NHANES) [27,28]. NHANES examined a nationally representative sample of the civilian, noninstitutionalized population of the US. Noninstitutionalized population constitutes of the individuals living in their homes (who are not the residents of any institutions such as prisons, skilled nursing facilities, mental facilities, etc.) NHANES paused the administration of balance questionnaire on the study participants after the 2003–2004 data cycle. It resumed the collection of this data on balance problems in 2019, but it was paused again due to the COVID-19 pandemic [29]. Due to these reasons, the current study had to rely on NHANES 2001–2004 data. In the absence of newer datasets containing the variables of interest, historical datasets can serve as a critical starting point, especially in emerging areas of inquiry [30]. This study was exempt from Institutional Review Board approval as it used the deidentified and publicly available data from NHANES.

The NHANES sample was selected using a four-stage process which began with the selection of Primary Sampling Units (PSUs). PSUs were counties or small groups of contiguous counties [27]. A block or a group of blocks within a PSU containing a cluster of households were determined as segments [27]. Households were randomly chosen from these segments and then one or more participants were selected from the households [27]. Trained interviewers administered the NHANES questionnaires in the participant’s household. Additional details about the survey design, sample selection, and informed consent used by NHANES are available elsewhere [27,28].

This study was conducted from March 2023 to August 2024. Analyses were carried out using demographic and standing balance questionnaire data from NHANES 2001–2002 and 2003–2004 cycles. These cycles were selected for this study because they were the latest cycles of nationally representative NHANES data for the US population aged 40 years and older that administered a balance questionnaire, available during the study period. NHANES data used in this study included an over-sampling of low-income individuals, those above 60 years of age, African Americans, and Mexican Americans [27,28]. This approach ensured that these groups were well-modeled in the data.

Participants were asked “During the past 12 months, have you had dizziness, difficulty with balance or difficulty with falling?” (hereafter, referred to as the “balance status” question). The available options were “yes”, “no”, “refused” or “don’t know”. Participants’ response to this question was used to ascertain the presence of balance problems for this study. Notably, NHANES grouped together dizziness, difficulty with balance or falling into a single variable in this question. While incongruencies between self-reported and performance-based measures have been recently reported [31] and dizziness may result from causes other than balance deficits, previous studies have used this question to identify the presence of self-reported balance problems [31,32,33]. Participants were further asked “Have you ever been treated by a doctor or other health (care) professional for dizziness, a balance problem, or falling?”. Similar response options of “yes”, “no”, “refused” or “don’t know” were available. All but eight of the study participants provided a “yes” or “no” response to this question about healthcare utilization for balance problems; one participant refused to answer and seven responded “don’t know”.

### 3.3. Participants

**Figure 1:**
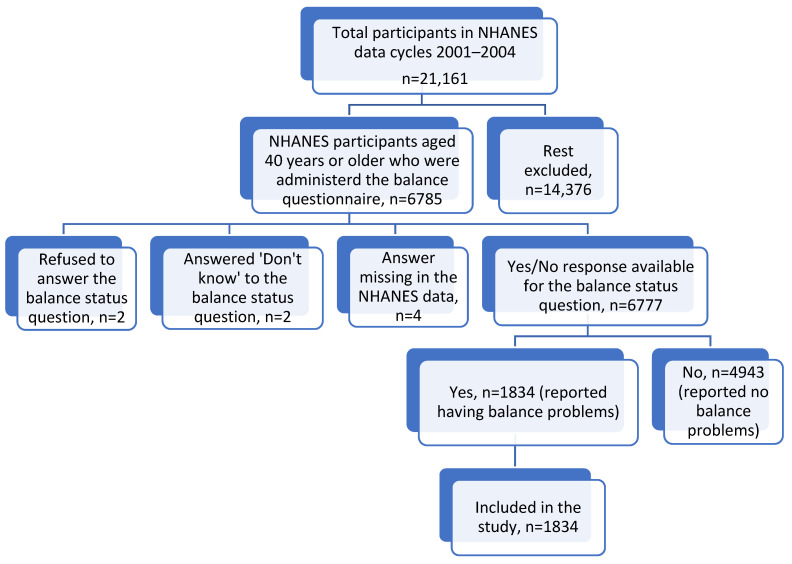
Flowchart detailing the inclusion criteria for study participants.

To study healthcare utilization for balance problems in community-dwelling adults and the factors associated with it, all adult individuals, aged 40 years or more, who self-reported having balance problems in the past 12 months, were included in the study. Of the total of 21,161 participants included in NHANES 2001–2002 and 2003–2004 data cycles, 6785 individuals were administered the balance status question. Individuals who answered ‘yes’ to this question, reflecting self-reported presence of balance problems in the past 12 months, were included in the study. The details of the inclusion of study participants are described in Figure 1.

### 3.4. Dependent Variable and Covariates

The dependent variable was a binary variable that assessed whether an individual ever saw a doctor or other healthcare professional for dizziness, a balance problem, or falling (referred to as healthcare utilization for balance problems). Predictors were drawn from the NHANES data as guided by the Andersen Healthcare Utilization Model [9,10], based on the literature review conducted to identify the potential factors [12]. The model included variables from all the three categories described by the Andersen Healthcare Utilization Model. Predisposing variables included in the model were age, sex, race, education level, marital status, number of comorbidities present (arthritis, congestive heart failure, coronary artery disease, stroke, cancer, diabetes mellitus), whether the individual saw a mental healthcare provider in the past year for mental health issues, and type of work/occupation. Enabling and disabling factors included in the model were annual family income, health insurance coverage, and whether the individual had any encounter(s) with healthcare provider (for any reason) over the past year or not. Variables affecting need for care included in the model were occurrence of fall(s) in the past year, general health status, and average level of physical activity. Age was the only continuous variable, and the rest were binary or categorical variables. Individuals aged 85 years or more were top coded at 85 years of age by NHANES [27]. The only variable identified by the literature review [12] which could not be included in this study due to the lack of availability for majority of the study participants was body mass index. The detailed description of all the variables used in this study is provided in Supplementary Materials.

### 3.5. Statistics

Data from NHANES 2001–2002 and 2003–2004 cycles was combined and probability weights were calculated as per the National Center for Health Statistics guidelines [34]. All analyses were adjusted using probability sampling weights [35,36] with measures of frequency, central tendency, and dispersion calculated for continuous and categorical variables as appropriate. An unadjusted relative association measure (Odds ratio, OR) and 95% confidence interval (95% CI) was calculated for each association using simple logistic regression [37]. An adjusted odds ratio was calculated using multivariable binary logistic regression. The multivariable binary logistic regression model [37] was used to explore the associations between different independent variables and the dependent variable, healthcare utilization for balance problems. Model fitness was evaluated using the Archer-Lemeshow goodness of fit test, which is a statistical test used to assess the fit of logistic regression models, particularly when dealing with probability sampling weights and complex survey designs [38]. None of the variables included in the multivariable regression model were missing more than 5% of the data, so no imputations were made for the missing data [39].

The multivariable logistic regression model (referred to as regular adjusted multivariable logistic regression model) was constructed using a combination of statistical (*p* < 0.1) and theoretical approaches based on literature review [12]. A recent literature review [12] indicated a possibility that the association between healthcare utilization for balance problems with the factors such as age, occurrence of falls and comorbidities could be modified by sex. Hence, age-sex interaction, sex-occurrence of fall(s) interaction, and sex-comorbidities interactions, in the context of healthcare utilization for balance problems, were investigated. None of these interactions was found to be statistically significant. Hence, there was no statistical basis to pursue any subgroup analyses. We also checked for multicollinearity among the independent variables using variance inflation factor (VIF) [40]. The mean VIF was 1.61, indicating no problem of multicollinearity.

To increase the interpretability of the study findings for clinicians and interdisciplinary readers, predicted probabilities of healthcare utilization for balance problems were also calculated for all the statistically significant predictor variables. For age, we calculated predicted probabilities of healthcare utilization at ten-year increments, that is, 40, 50, 60, 70, and 80 years of age.

To compare the relative magnitude of the associations of different explanatory variables with the dependent variable, based on the same measurement scale, the independent variables were standardized or scaled. This was based on Gelman et al.’s recommendation that continuous variables should be divided by two times the standard deviation instead of one to match the scale of binary variables [41]. Only the continuous variables were standardized and binary variables were left as is, as proposed by literature on scaling regression inputs [41]. Standardized age variable was created by using the following equation:

Standardized age = [Age − (Mean of age)]/2(Standard deviation of age)

Standardized age (along with all the other predictor variables) was used to construct a standardized multivariable regression model. The statistical analysis was conducted using the STATA version 18.0 software (StataCorp., College Station, TX, USA). A two-tailed *p*-value of 0.05 was considered significant.

## 4. Results

### 4.1. Baseline Characteristics of Individuals with Self-Reported Balance Problems

A total of 1834 individuals, who self-reported having balance problems in the past 12 months were included in this study. The baseline characteristics of the individuals who self-reported having balance problems have been detailed in Table 1. Of the 1834 individuals, the mean age was 60.1 years (0.5 standard error; range 40 to 85 years) and 62.26% were females. With respect to race, 76.77% were Non-Hispanic Whites, with 9.37% being Non-Hispanic Blacks, 8.81% being Hispanics, and 5.05% belonging to other races. An estimated 45.35% had more than high school education, 28.27% had high school education and 26.37% of the participants had less than high school education. Majority of the participants (58.81%) were either married or living with a partner. The annual family income was above $20,000 for 65.29% of the participants.

Of all the individuals with self-reported balance problems, 25.84% reported having one or more falls in the past year (Table 1) and 33.48% had no other comorbidity. An estimated 93.38% of individuals reported encounter(s) with a healthcare provider (for any reason) over the past year. A total of 11.77% of those with balance problems did not have health insurance. Further details about the baseline characteristics of the study population are provided in Table 1.

### 4.2. Healthcare Utilization for Balance Problems

Only 32.13% of the individuals (aged 40 years or more) who self-reported having balance problems ever sought healthcare services for balance problems. The results of unadjusted simple logistic regression are detailed in Table 2. Annual family income, health insurance coverage, encounter(s) with healthcare provider in the past year (for any reason), and falls in the past year had statistically significant associations with healthcare utilization for balance problems, in the unadjusted model. Individuals whose annual family income was more than $20,000 had 1.43 times higher odds of using healthcare for balance problems (OR 1.43; 95% CI, 1.02–2.00; *p* = 0.04). Those without health insurance coverage had 59% reduced odds of healthcare utilization for their balance problems (OR 0.41; 95% CI, 0.26–0.63; *p* < 0.001). Individuals who had seen a healthcare provider for any reason in the past year were 3.26 times more likely to seek healthcare services for their balance problems (OR 3.26; 95% CI, 1.56–6.78; *p* = 0.003). Individuals with self-reported balance problems who experienced falls in the past year had 35% increased odds of using healthcare for their balance problems (OR 1.35; 95% CI, 1.05–1.73; *p* = 0.02).

### 4.3. Predictors of Healthcare Utilization for Balance Problems—Results of the Regular Adjusted Multivariable Logistic Regression Model

The regular adjusted multivariable logistic regression results show that age, encounter(s) with a healthcare provider in the past year for any reason, health insurance, and falls in the past year were all significant predictors of healthcare utilization for balance problems. For every one-year increase in age, the odds of seeking healthcare services for balance problems reduced by 2% (AOR, 0.98; 95% CI, 0.97–0.996; *p* = 0.011). Seeing a healthcare provider in the past year for any reason was associated with 2.45 times increased odds of using healthcare services for balance problems (AOR 2.45; 95% CI, 1.19–5.06; *p* = 0.017). Not having health insurance coverage was associated with 48.4% decrease in the likelihood of using healthcare services for the management of balance problems (AOR 0.52; 95% CI, 0.32–0.84; *p* = 0.009). Falls in the past year significantly increased the odds of healthcare utilization for balance problems by 28.8% (AOR, 1.29; 95% CI, 1.03–1.61; *p* = 0.028). The detailed results of the regular adjusted multivariable logistic regression model with adjusted odds ratios and 95% confidence intervals are provided in Table 3.

### 4.4. Predicted Probabilities of Healthcare Utilization for Balance Problems

**Figure 2:**
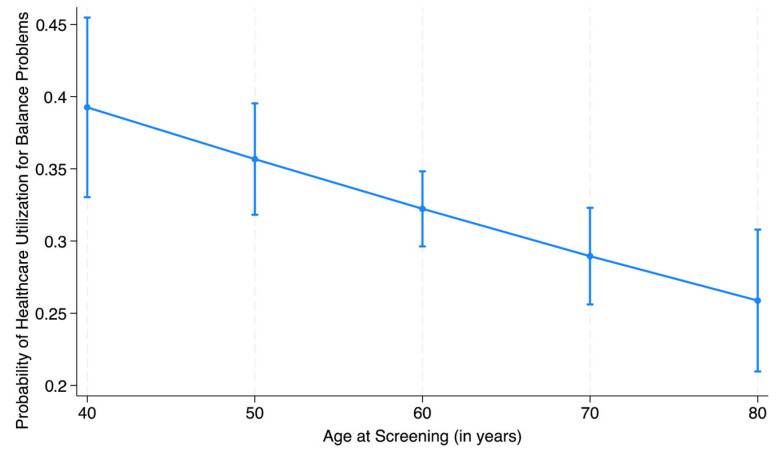
Predicted probability of healthcare utilization for balance problem with advancing age (with 95% confidence intervals).

To enhance the interpretability of the findings for a broader audience and provide additional understanding of the strength of effect estimates relative to each other, predicted probabilities of healthcare utilization are reported for the significant predictors. The probability of healthcare utilization for balance problems in community-dwelling adults reduced with increasing age, as shown in Figure 2, when all the other factors were kept constant. The probability of seeking healthcare services for balance problems reduced from 0.39 for a 40-year-old with self-reported balance problems to 0.26 for an 80-year-old with similar problems. Those who reported falling in the past year had a 0.36 probability for seeking healthcare services for balance problems compared to 0.31 in those who did not experience any falls. The probability of healthcare utilization was almost twice for individuals who saw a healthcare provider in the past year compared to those who did not (0.33 vs. 0.17). Lastly, the predicted probability of using healthcare for balance problems was 0.33 among those with health insurance coverage compared to only 0.21 among those who did not have insurance, keeping all the other factors constant.

### 4.5. Results of the Standardized Multivariable Logistic Regression Model

Standardization of age was performed to compare the relative magnitude of the odds ratios of different explanatory variables with healthcare utilization for balance problems. Based on the standardized coefficients obtained from the standardized regression model, seeing a healthcare provider in the past year for any reason had the relatively largest influence on healthcare utilization (AOR = 2.45; 95% CI, 1.19–5.06; *p*-value = 0.02), followed by the impact of health insurance coverage (AOR = 0.52; 95% CI, 0.32–0.84; *p*-value < 0.01), age (AOR 0.64; 95% CI, 0.45–0.89; *p*-value = 0.01), and falls in the past year (AOR = 1.29; 95% CI, 1.03–1.61; *p*-value = 0.03).

## 5. Discussion

This study reports the factors associated with reduced healthcare utilization for balance problems in a nationally representative sample of community-dwelling adults with self-reported balance problems in the US. Only 32.13% of individuals with balance problems sought healthcare services to address their balance problems. Older age and lack of health insurance were statistically significant predictors of reduced healthcare utilization for balance problems. Additionally, individuals who reported no encounter(s) with healthcare provider for any reason in the past year had reduced odds of seeking healthcare services for their balance problems. Lastly, individuals who had not experienced any falls in the past year were less likely to seek healthcare services for their balance problems, indicating that people probably tend to wait till they start falling before seeking any help.

This study reports a 2% reduction in the odds of seeking healthcare for balance problems with every one-year increase in age. The probability of seeking healthcare services for balance problems for an 80-year-old with self-reported balance problems was reduced to about two-thirds of the probability for a 40-year-old with similar problems, when all the other factors were kept constant. Foley et al. reported that older adults usually have a higher overall utilization of healthcare services compared to younger adults [42]. However, it has also been reported that older adults, though more likely to seek healthcare services for chronic problems or pre/post-surgery, are less likely to seek preventive services [43]. This aligns with the decreasing trend for healthcare utilization for balance problems with increasing age as reported by our study. Hence allocation of resources towards educating the elderly individuals about the availability of healthcare services, that can potentially help improve their balance and prevent the possibilities of serious events or falls, is warranted. Efforts may also be needed to identify and address factors, such as transportation issues and mobility issues in the elderly, which may interfere with an individual’s access to healthcare services, as reported by Clarke [44].

Our study also reports that community-dwelling adults with balance problems had 145% greater odds of seeking healthcare services for their balance problems if they had encounter(s) with healthcare provider for any reason in the past year. This finding aligns with the existing literature reporting worse health outcomes for individuals living in medically underserved areas with limited access to healthcare services [45]. Healthcare visits, particularly primary care visits increase the likelihood of preventive interventions [46]. Individuals with balance problems are likely getting referred for fall prevention interventions during their regular healthcare visits. Implementation of initiatives such as the ‘Stopping Elderly Accidents, Deaths, and Injuries’ (STEADI) initiative to incorporate fall risk assessment into routine clinical practice [47] would further enhance this association between healthcare utilization for balance problems and seeing a healthcare provider in the past year. The study findings support the STEADI initiative and underscore the importance of discussing balance problems during the visit with a healthcare provider for any reason, as it could likely promote healthcare utilization for balance problems. Age-related differences have been reported in patient perceptions of the encounter with healthcare providers [48], as well their health outcomes after the encounter [49]. Future research should explore the quality of encounter with the healthcare provider for any reason, as it may have an impact on healthcare utilization for balance problems.

Health insurance had the second largest relative impact on healthcare utilization for balance problems in our study, as shown by the results of the standardized multivariable regression model. The odds of healthcare utilization for balance problems were nearly 50% less for the individuals who lacked health insurance compared to those who had it. Lack of health insurance coverage is a crucial driver of the disparities seen in healthcare [50]. Health insurance coverage increases the individual’s likelihood to seek preventive and clinical services [50,51,52]. A universal health coverage in the US could likely increase timely management of balance problems and possibly reduce the burden associated with falls and related injuries, especially for the individuals that lack health insurance in the current system.

Participants in our study who reported fall(s) in the past year had 35% greater odds of seeking healthcare services for their balance problems. These findings imply that individuals with self-reported balance problems are less likely to seek preventive measures if they have not experienced any fall(s) in the past year. However, any fall can be debilitating and may result in serious injuries [2,8]. Hence, education regarding the availability of effective multi-factorial fall prevention interventions should be considered for those with self-reported balance problems, even if they have not experienced any falls yet. Multimodal fall prevention approaches led by physical therapists [53] and community-based interventions [54] have been reported to reduce falls and fall-related injuries, especially in community-dwelling older adults. A study done in Australia by Beard et al., demonstrated the overall benefit to cost ratio for a multimodal community-based fall intervention to be around 21:1, with a 20% reduction in the fall-related hospital admissions [55]. A special report from United States Centers for Disease Control and Prevention detailed positive benefits and return of investment ranging from 36% to 509% for different community based fall interventions [56]. These findings suggest that the identification and removal of the barriers to healthcare utilization for balance problems can not only improve the patient outcomes, but also reduce the economic burden on the healthcare system from fall-related injuries.

This study has several notable strengths. Use of nationally representative data provides a generalizable starting point for research in this domain [30]. The application of the Andersen Healthcare Utilization Model provided a strong theoretical framework [12], while robust methodology—including multivariable logistic regression, adjustments for probability sampling weights, and testing for interactions—ensured analytic rigor. Importantly, the study highlights the impact of health insurance, age, contact with a healthcare professional in the past year and prior falls on healthcare utilization for balance problems. It provides useful information for hypothesis generation for further research in the area of health service utilization for balance problems in the community-dwelling population of the US. However, some limitations warrant attention. This study used NHANES data from 2001 to 2004. Unfortunately, more recent NHANES data cycles available at the time of study lacked the balance information variables of interest. Future research can use these preliminary results on more recent data, as it becomes available. Furthermore, these findings can inform the design of prospective studies exploring healthcare utilization for balance problems. Additionally, NHANES data collected self-reported information on individuals’ sex through a question labeled as gender, with response options being male or female [27,57]. Sex and gender are distinct constructs [57]; however, in the 2001–2004 NHANES data cycles, these terms were often conflated due to limited differentiation between the two at the time. Furthermore, given the use of self-reported information in this study, the presence of bias related to recall or the tendency to provide socially desirable responses cannot be negated. While oversampling of certain groups by NHANES ensured that these populations are well-represented and accurately modeled in the data, it could lead to increased variance in overall estimates [58]. However, in this study, the large sample size and the application of advanced statistical techniques mitigate this concern.

Access to care is an important factor that can drive healthcare utilization. While the current study included variables to account for socio-economic status and health insurance coverage, we could not look at the geographic factors such as living in a rural vs. urban community, distance from the nearest hospital/healthcare provider, etc. Future studies should consider including more information about the accessibility to care in their models. Additionally, the dependent variable in the current study was binary which assessed whether an individual ever saw a healthcare professional for their balance problems or not. We do acknowledge that those with balance problems might have engaged in non-traditional ways of trying to address their balance deficits, for example, by engaging in community-based fall prevention interventions, community exercise classes, such as joining Pilates or tai chi, which could not be captured by the available data. Also, the available data did not allow for differentiation between one visit versus multiple visits that might be needed for successful management of balance deficits. Perhaps a model looking at the continuum of care can be more helpful in understanding the complete picture of healthcare utilization for balance problems.

## 6. Conclusions

This study reports the underutilization of healthcare services for the management of balance problems in community-dwelling adults with self-reported balance problems. Older age, lack of health insurance, not seeing a healthcare provider in the past year, and not experiencing any falls in the past year had a significant association with reduced healthcare utilization for balance problems. These findings identify the populations at potential risk of underutilizing healthcare services for balance problems and might be useful in guiding the efforts towards an increased and equitable use of effective fall prevention interventions for all individuals with balance problems. However, this study relied on older data and future research using the newer data is warranted to validate and expand these findings.

## Figures and Tables

**Table 1:** Basalance problems.

Characteristics	Weighted Mean (Weighted Standard Error)	Frequency (Weighted %) *
**Baseline characteristics**		
**Age, years (n = 1834)**	60.08 (0.46)	
**Sex (n= 1834)**		
**Female**		1091 (62.26)
**Male**		743 (37.74)
**Race (n = 1834)**		
**Non-Hispanics Whites**		1115 (76.77)
**Mexican American/other Hispanics**		370 (8.81)
**Non-Hispanics Blacks**		283 (9.37)
**Others**		66 (5.05)
**Education (n = 1822)**		
**Less than high school**		693 (26.37)
**High school**		462 (28.27)
**More than high school**		667 (45.35)
**Marital status (n = 1832)**		
**Married/living with partner**		973 (58.81)
**Widowed/Divorced/separated**		749 (34.74)
**Never married**		110 (6.46)
**Annual family income (n = 1767)**		
**Less than $20,000**		789 (34.71)
**$20,000 or more**		978 (65.29)
**Health insurance (n = 1814)**		
**Yes**		1617 (88.23)
**No**		197 (11.77)
**Type of work (n = 1832)**		
**Not working at job or business**		1337 (60.27)
**Working at job or business**		447 (35.17)
**With job or business but not working past week**		33 (3.03)
**Looking for work**		15 (1.52)
**Number of total comorbidities (n = 1834)**		
**0**		512 (33.48)
**1**		687 (36.53)
**2**		384 (18.46)
**3**		179 (8.31)
**4**		59 (2.8)
**5**		11 (0.36)
**6**		2 (0.067)
**Mental health issues/seeing mental health provider (n = 1833)**		
**No**		1615 (85.51)
**Yes**		218 (14.49)
**Encounter(s) with healthcare provider in the past 1 year for any reason (n = 1833)**		
**No**		116 (6.62)
**Yes**		1717 (93.38)
**Physical activity (n = 1827)**		
**Sedentary (mostly sits, not walk much)**		742 (36.75)
**Stands/walks but no carrying/lifting**		847 (46.89)
**Lifts light load, climbs stairs often**		179 (11.8)
**Heavy work**		59 (4.56)
**General Health (n = 1834)**		
**Excellent**		120 (7.46)
**Very good**		306 (20.23)
**Good**		548 (32.66)
**Fair**		573 (25.6)
**Poor**		287 (14.06)
**Falls in the past year (n = 1832)**		
**Yes**		537 (25.84)
**No**		1295 (74.16)

* Frequency reflects the actual observed sample; while the weighted % is the percentage of population calculated using probability sampling weights to obtain nationally representative characteristics.

**Table 2:** Crude/unadjusted odds ratios of the relationship between healthcare utilization for balance problems and different covariates (Results of Bivariate simple logistic regression).

Covariate	Unadjusted OR (95% CI)	*p*-Value
**Age**	0.997 (0.99–1.01)	0.55
**Sex**		
**Male**	Reference	
**Female**	0.91 (0.69–1.20)	0.49
**Race**		
**Non-Hispanics Whites**	Reference	
**Mexican American/other Hispanics**	0.74 (0.49–1.12)	0.15
**Non-Hispanics Blacks**	1.09 (0.88–1.36)	0.41
**Others**	0.63 (0.28–1.38)	0.24
**Education**		
**Less than high school**	Reference	
**High school**	1.09 (0.74–1.62)	0.63
**More than high school**	1.27(0.94–1.73)	0.12
**Marital status**		
**Married/living with partner**	Reference	
**Widowed/Divorced/separated**	0.78 (0.60–1.02)	0.07
**Never married**	0.59(0.24–1.43)	0.23
**Annual family income**		
**Less than $20,000**	Reference	
**$20,000 or more**	**1.43 (1.02–2.00)**	**0.04**
**Health insurance**		
**Yes**	Reference	
**No**	0.41 (0.26–0.63)	**<0.001**
**Type of work**		
**Not working at job or business**	Reference	
**Working at job or business**	0.87 (0.60–1.24)	0.43
**With job or business but not working past week**	0.89 (0.31–2.56)	0.83
**Looking for work**	0.40 (0.14–1.16)	0.09
**Number of total comorbidities**		
**0**	Reference	
**1**	1.31 (0.96–1.78)	0.09
**2**	1.32 (0.90–1.93)	0.16
**3**	1.58 (1.01–2.46)	0.05
**4**	1.45 (0.68–3.07)	0.33
**5**	3.22 (0.71–14.70)	0.13
**6**	2.79 (0.17–47.08)	0.46
**Mental health issues/seeing mental health provider**		
**No**	Reference	
**Yes**	1.02 (0.74–1.39)	0.10
**Encounter(s) with healthcare provider in the past year for any reason**		
**No**	Reference	
**Yes**	**3.26 (1.56–6.78)**	**0.003**
**Physical activity**		
**Sedentary (mostly sits, not walk much)**	Reference	
**Stands/walks but no carrying/lifting**	0.86 (0.66–1.13)	0.28
**Lifts light load, climbs stairs often**	0.91 (0.65–1.28)	0.58
**Heavy work**	0.71 (0.31–1.63)	0.40
**General Health**		
**Excellent**	Reference	
**Very good**	1.19 (0.63–2.26)	0.58
**Good**	1.06 (0.57–1.99)	0.84
**Fair**	1.07 (0.62–1.83)	0.81
**Poor**	1.50 (0.82–2.75)	0.18
**Falls in the past year**		
**No**	Reference	
**Yes**	**1.35 (1.05–1.73)**	**0.02**

Statistically significant values are in bold.

**Table 3:** Significant predictors of healthcare utilization for balance problems in the regular adjusted multivariable logistic regression model (n = 1745).

Covariate	Adjusted OR (95% CI)	*p*-Value
Age	0.98 (0.97–0.996)	0.01
Falls in the past year	1.29 (1.03–1.61)	0.03
Encounter(s) with healthcare provider in past year for any reason	2.45 (1.19–5.06)	0.02
Lack of health insurance	0.52 (0.32–0.84)	<0.01

Archer Lemeshow Goodness of fit test for this multivariable regression; *p*-value = 0.65. Variables included in the model: age, sex, race, education level, marital status, annual family income, occurrence of fall(s) in the past year, number of comorbidities present, whether seeing a mental healthcare provider for mental health issues, general health status, whether covered by health insurance or not, whether received healthcare services over the past year or not, type of work/occupation, and average level of physical activity.

## Data Availability

The original data presented in the study are openly available in [NHANES] at [https://www.cdc.gov/nchs/nhanes/index.html].

## Supplementary Materials

The following supporting information can be downloaded at: https://www.mdpi.com/article/10.3390/healthcare13202654/s1, Table S1: Additional information about the criterion used to obtain data on each of the variables (covariates) used in this study.
