# Supplementary material for: Factors Associated with Health Service Use for Self-Reported Balance Problems in Community-Dwelling Adults: A Secondary Analysis of Nationally Representative NHANES 2001–2004 Data
Source: Healthcare (Basel). 2025 Oct 21;13(20):2654. doi: 10.3390/healthcare13202654 (PMC12562649; doi:10.3390/healthcare13202654)
Supplement: Supplementary file 1 [file healthcare-13-02654-s001.zip › healthcare-3858383-supplementary.pdf]

Supplemental content

Table S1: Additional information about the criterion used to obtain data on each of the variables (covariates) used in this study

**Covariate**	**Criterion**
Age	Age at the time of screening interview (in years)
Sex	Biological sex (options male or female)
Race	Using the combined information about race and ethnicity. All Hispanic participants were grouped as Mexican American/ Hispanics. Non- Hispanic participants were categorized, based on self-reported race as Non- Hispanic Black, Non- Hispanic White and Others (which included non- Hispanics Multiracials)
Number of comorbidities	Comorbidities included: Arthritis, Congestive Heart failure, Coronary Artery disease, Stroke, Cancer, Diabetes Mellitus
Presence of mental health issues	Proxy used: Seen mental health provider in the past year
Health insurance	Whether covered by health insurance or some other kind of health care plan, including health insurance obtained

	through employment or purchased directly as well as government programs like Medicare and Medicaid
**Encounter(s) with a healthcare provider for any reason** in the past year	Self-reported answer to whether the individual saw a doctor or other health care professional about one's health at a doctor's office, a clinic, hospital emergency room, at home or some other place, for any reason, during the past 12 months
General Health Status	Self-reported answer to the question: How would say your general health is?
Occurrence of falls in the past year	Self-reported answer to a question about any falls in the past year. United States Center for Disease Control and Prevention (CDC) defines falls as unexpected or unintentional dropping to a lower surface (i.e., the floor), from a standing, seating, walking, or bending position. However, a formal definition of 'falls' was missing from the NHANES manuals
Physical Activity	Average level of physical activity during the daily activities of a typical day

	(including work, housework, going to and attending classes etc.)
Type of work	Self-reported information about current job/business, specifically type of work done in the week preceding the interview
Annual Family income	U.S. Bureau of the Census Current Population Survey definition of “family” was used to group household members into one or more families. Family was defined as: “a group of two people or more (one of whom is the householder) related by birth, marriage, or adoption and residing together”
Education	Highest grade or level of school completed or the highest degree
Marital status	Based on information obtained during NHANES household interview
